# Mitochondrial Abnormalities of T Lymphocytes in Patients With Maintenance Hemodialysis

**DOI:** 10.1002/iid3.70404

**Published:** 2026-03-17

**Authors:** Chunlei Luo, Sizeng Bao, Xueyan Bian, Lingling Bao, Chunyang Ji, Jianwei Ma, Jiancheng Huang

**Affiliations:** ^1^ Department of Nephrology The First Affiliated Hospital of Ningbo University Ningbo Zhejiang China

## Abstract

**Objective:**

To investigate the characteristics of T lymphocyte subsets and mitochondrial function in hemodialysis patients and to explore the effect of hemodialysis on these parameters.

**Methods:**

Patients with end‐stage renal disease (ESRD) from July 2023 to June 2024, who underwent mitochondrial function detection of T lymphocyte subsets, were included in this retrospective study. Patients were categorized into a hemodialysis group and a pre‐dialysis group based on whether they received hemodialysis. The hemodialysis group was further divided into those with dialysis duration ≤ 12 months and those with > 12 months. Peripheral blood from ESRD patients was analyzed by flow cytometry to obtain data on T lymphocyte subsets, mitochondrial mass (MM), and low mitochondrial membrane potential (MMP‐Low). Differences in T lymphocyte subsets mitochondrial parameters among distinct groups were analyzed, and the association between hemodialysis and the aforementioned mitochondrial parameters was investigated using multiple linear regression analysis.

**Results:**

A total of 250 patients were enrolled (153 hemodialysis and 97 pre‐dialysis). Compared with the pre‐dialysis group, the hemodialysis group had significantly lower white blood cell count (5.62 ± 1.63 vs. 6.49 ± 1.93 × 10^3^/mm^3^, *p* < 0.001) and neutrophil percentage (67.43 ± 8.10 vs. 70.73 ± 9.41%, *p* = 0.004), but a higher lymphocyte percentage (20.13 ± 6.39 vs. 17.73 ± 7.05%, *p* = 0.007). CD45+ lymphocyte percentage was also significantly higher in the hemodialysis group(20.13 ± 7.15 vs. 17.74 ± 7.28, *p* = 0.011), whereas the MM of CD3+, CD4+, and CD8+ T lymphocytes (2.66 ± 1.03 vs. 3.18 ± 1.45, *p* = 0.001; 2.96 ± 1.17 vs. 3.56 ± 1.53, *p* = 0.001; 2.28 ± 1.22 vs. 2.81 ± 1.64, *p* = 0.007, respectively), and the MMP‐Low% of these T lymphocytes (21.86 ± 10.60 vs. 26.97 ± 11.76, *p* = 0.001; 15.53 ± 9.78 vs. 20.66 ± 10.42, *p* < 0.001; 31.12 ± 13.25 vs. 35.28 ± 15.17, *p* = 0.024, respectively), were significantly reduced. Among hemodialysis patients, those with dialysis duration > 12 months had significantly lower CD4+ T lymphocytes (455.66 ± 232.80 vs. 375.06 ± 150.99, *p* = 0.029) and MMP‐Low% of CD3+ (25.17 ± 14.08 vs. 20.25 ± 8.02, *p* = 0.025), CD4+ (18.30 ± 13.47 vs. 14.18 ± 7.06, *p* = 0.046), and CD8+ (35.22 ± 16.09 vs. 29.13 ± 11.19, *p* = 0.019) T lymphocytes than those with ≤ 12 months. Linear regression analysis, after adjusting for multiple covariates, confirmed a significant association between hemodialysis and reduced mitochondrial parameters: decreased MM of CD3+ (0.699), CD4+ (0.735) and CD8+ (0.846) T lymphocytes, and reduced MMP‐Low% of CD3+ (4.374%) and CD4+ (4.630%) T lymphocytes.

**Conclusion:**

T lymphocyte MM and MMP‐Low% differ significantly between hemodialysis and pre‐dialysis ESRD patients, and hemodialysis patients exhibit more pronounced T lymphocyte mitochondrial dysfunction.

## 1 Introduction

In patients with end‐stage renal disease (ESRD), severe metabolic disorders disrupt the delicate equilibrium between immune activation and suppression [1], resulting in systemic immune dysfunction. Emerging evidence indicates this immunopathology stems from three interrelated mechanisms: (1) Accumulation of protein‐bound uremic toxins (particularly p‐cresol sulfate and indoxyl sulfate) interferes with T‐cell receptor signaling pathways and mitochondrial electron transport chain function; (2) A chronic inflammatory milieu characterized by elevated IL‐6, TNF‐α, and CRP levels drives T‐cell exhaustion through persistent immune activation; (3) Metabolic acidosis‐induced alterations in cellular redox state (NAD + /NADH ratio) impair mitochondrial bioenergetics in lymphocytes. These pathological changes manifest clinically as multiple immune defects, including impaired phagocytic activity of myeloid cells, deficient antigen presentation, diminished B‐cell antibody production, and compromised T‐cell mediated immunity [2].

As the central orchestrators of adaptive immunity, T lymphocytes exhibit particularly pronounced abnormalities in ESRD. It has been confirmed that lymphocyte counts, including CD4+ T lymphocytes and CD8+ T lymphocyte subsets, are significantly reduced in ESRD patients [3], and low lymphocyte levels are closely associated with an increased risk of mortality [4]. Mitochondrial transfer from damaged cells may exacerbate these deficits, as observed in other chronic inflammatory conditions where acquired mitochondrial abnormalities induce T cell senescence.

Mitochondria are highly dynamic organelles responsible for synthesizing adenosine triphosphate (ATP), regulating metabolism, producing reactive oxygen species (ROS), and controlling cell differentiation and death [5]. They play a central role in T cell energy metabolism and cell homeostasis and are essential for inducing and regulating immune responses [6]. Mitochondrial dysfunction is linked to oxidative stress and increased inflammation [7]. Although earlier studies have documented mitochondrial abnormalities in peripheral blood mononuclear cells (PBMCs) of patients with chronic kidney disease (CKD) [8, 9], the specific impacts of hemodialysis on T lymphocyte mitochondrial parameters remain poorly characterized. Our retrospective cohort study addresses this knowledge gap by systematically comparing T lymphocyte subset distribution and mitochondrial function profiles between pre‐dialysis and maintenance hemodialysis ESRD patients, providing novel insights into the effects of renal replacement therapy on the immune system.

## 2 Materials and Methods

### 2.1 Patients

Patients with ESRD who were either under outpatient follow‐up or hospitalized at the First Affiliated Hospital of Ningbo University from July 2023 to June 2024 were enrolled. Inclusion criteria were: (1) age ≥ 18 years; (2) diagnosis of ESRD according to KDIGO 2012 Clinical Practice Guidelines (creatine based estimated glomerular filtration rate < 15 ml/min/1.73 m^2^, or requirement for renal replacement therapy), including both pre‐dialysis (ESRD patients who had not yet initiated renal replacement therapy, all managed comprehensively with conservative care, and under regular nephrology follow‐up with anticipated dialysis initiation within 6–12 months based on disease progression.) and hemodialysis patients; (3) availability of T lymphocyte mitochondrial function test results. Exclusion criteria included: (1) patients receiving peritoneal dialysis or renal transplantation; (2) patients with acute symptomatic infections, active immune diseases, malignant diseases, and pregnant. Patients were categorized into a hemodialysis group and a pre‐dialysis group based on whether they were undergoing hemodialysis. Hemodialysis patients were further stratified into two groups using a 12‐month cutoff (≤ 12 months and > 12 months) to distinguish between early‐phase and long‐term hemodialysis patients. This was a cross‐sectional study and was approved by the hospital Ethics Committee (2025 Research No. 205RS). No interventions were implemented during the study that would affect patient welfare, and an exemption from informed consent was granted and approved.

### 2.2 Data Collection

Demographic data, including gender, age, height, weight, and underlying conditions, were collected, along with dialysis duration, the hemodialysis regimen, type of dialyzer, dialysis solution, Kt/V, number of weekly sessions, and duration of session for hemodialysis patients.

For pre‐dialysis patients, fasting venous blood samples were collected directly, while for hemodialysis patients, blood samples were collected immediately before the start of the first weekly hemodialysis session (typically Monday/Tuesday morning), with patients in a fasting state. The blood samples of all the subjects were transported to the central laboratory of the hospital immediately for laboratory analysis. Laboratory data were recorded, including high‐sensitivity C‐reactive protein, white blood cell count, neutrophil percentage, lymphocyte percentage, hemoglobin, platelet count, serum albumin, globulin, urea nitrogen, creatinine, uric acid, high‐density lipoprotein cholesterol, low‐density lipoprotein cholesterol, blood calcium, and phosphorus. Body Mass Index (BMI) was calculated. Biochemical parameters were detected by the Beckman Coulter AU 5800 (Beckman Coulter Inc., Brea, CA, USA), and Hematological parameters were analyzed using the Sysmex XN‐10.

### 2.3 Laboratory Testing

Mitochondrial function of T lymphocytes in peripheral blood was assessed using flow cytometry. Parameters measured included CD45+ lymphocytes, CD3+, CD4+, and CD8+ T lymphocytes, mitochondrial mass (MM), and low mitochondrial membrane potential (MMP‐Low). The experimental operation method obtained is as follows: (a) The peripheral blood sample was collected in tubes coated with EDTA‐k anticoagulant and detected within 48 h. (b) Add the mixture antibody and sample, staining for 30 min at room temperature. (c) Mixing and MitoDye staining at 37°C for 30 min. (d) The parameters were detected by flow cytometer. These data were calibrated by the human lymphocyte mitochondrial function analysis system (software) and were output. The reagent used was the CD8 + 19FITC/CD3 + 56PE/CD45PC5.5/CD4PC7/MitoDye detection kit (UB32229‐8, Ubipeptide Biotechnology (Zhejiang) Co., LTD.). MM was calculated by the median fluorescence index of mitochondria. MMP‐Low%, the percentage of MMP low population cells by flow cytometry, was clustered by mitochondrial specific marker MitoDye and gated the percentage of low population cells (ratio value).

### 2.4 Statistical Analysis

Statistical analysis was performed using SPSS 26.0 software. The Shapiro‐Wilk test and Q–Q plot were used to assess the normality of the data. Normally distributed variables were expressed as mean ± standard deviation, and comparisons between groups were made using the T‐test (Levene's test confirmed the homogeneity of variance) and the Welch *t*‐test (non‐homogeneous variances). For these continuous outcome comparisons, effect sizes were quantified using Cohen's *d*. Variables with non‐normal distribution were expressed as median (P25, P75), and the permutation test was used for group comparisons. Categorical data were expressed as percentages, and the Chi‐square test was used for comparisons; the strength of association was measured by Cramer's V as the effect size indicator. Effect sizes were interpreted according to conventional thresholds (Cohen, 1988): small effect (Cohen's *d* = 0.2; Cramer's *V* = 0.1), medium effect (Cohen's *d *= 0.5; Cramer's *V* = 0.3), and large effect (Cohen's *d* = 0.8; Cramer's *V* = 0.5).

To evaluate the effect of hemodialysis on mitochondrial parameters in T lymphocytes, multiple linear regression models were constructed. The dependent variables were continuous mitochondrial parameters of T lymphocytes. The selection of independent variables was primarily based on clinical judgment derived from the study's clinical context: variables were included if they were clinically recognized as potential confounders or correlates of T lymphocyte mitochondrial function (e.g., demographic factors [gender, age], metabolic indicators [BMI, low‐density lipoprotein cholesterol], comorbidities [diabetes mellitus], and hematological parameters [white blood cell count, hemoglobin, albumin])—consistent with clinical practice and prior research implications in related fields. Stepwise regression (forward selection with probability of F‐to‐enter ≤ 0.05 and F‐to‐remove ≥ 0.10) was used to screen predictive factors, balancing model parsimony and explanatory power. For the regression models, the adjusted *R* ^2^ was reported to indicate the proportion of variance in the dependent variable explained by the final model (a supplementary measure of effect size for predictive analyses). Prior to model fitting, all key assumptions of multiple linear regression were verified: (1) Linearity between independent variables and dependent variables was confirmed via scatter plots of standardized residuals versus predicted values; (2) Independence of observations was validated by Durbin‐Watson (DW) test, with values close to 2 indicating no autocorrelation; (3) Homoscedasticity was assessed via residual plots, showing no systematic patterns of residual variance; (4) Absence of multicollinearity was confirmed by variance inflation factors (VIF) < 2 for all variables; (5) Normality of residual distribution was verified using Shapiro‐Wilk test and Q–Q plots, with no significant deviations from normality (all *p* > 0.05). All statistical tests were bilateral, and *p* < 0.05 was considered statistically significant.

## 3 Results

### 3.1 Demographic and Laboratory Data

A total of 250 patients were enrolled in this study, including 154 males and 96 females, with a mean age of 63.6 ± 15.1 years. The cohort included 153 patients in the hemodialysis group and 97 patients in the pre‐dialysis group. A comparison of the demographic and laboratory data between the two groups is presented in Table 1. Compared with the pre‐dialysis group, the BMI of patients in the hemodialysis group was relatively lower (22.36 ± 3.14 vs. 23.77 ± 3.64 kg/m^2^, *p* = 0.005, Cohen's *d* = −0.771); however, there were no significant differences in gender, age, or the prevalence of hypertension and diabetes. Laboratory indicators showed that the white blood cell count (5.62 ± 1.63 vs. 6.49 ± 1.93 × 10^3^/mm^3^, *p* < 0.001, Cohen's *d* = ‐0.659), neutrophil percentage (67.43 ± 8.10 vs. 70.73 ± 9.41%, *p* = 0.004, Cohen's *d* = −1.123), platelet count (169.37 ± 61.55 vs. 192.88 ± 76.12 × 10^3^/mm^3^, *p* = 0.008, Cohen's *d* = −2.869), uric acid (397.35 ± 101.85 vs. 438.16 ± 152.08 μmol/L, *p* = 0.021, Cohen's *d* = −3.705), total cholesterol (3.45 ± 1.01 vs. 4.18 ± 1.46 mmol/L, *p* < 0.001, Cohen's *d* = −0.667), and low‐density lipoprotein cholesterol (2.16 ± 0.74 vs. 2.62 ± 1.03 mmol/L, *p* < 0.001, Cohen's *d* = −0.493) were lower in the hemodialysis group. In contrast, lymphocyte percentage (20.13 ± 6.39 vs. 17.73 ± 7.05%, *p* = 0.007, Cohen's *d* = 0.931), hemoglobin (109.54 ± 15.17 vs. 87.38 ± 22.44 g/L, *p* < 0.001, Cohen's *d* = 5.225), albumin (36.81 ± 4.34 vs. 33.53 ± 5.16 g/L, *p* < 0.001, Cohen's *d* = 1.524), creatinine (810.35 ± 251.91 vs. 607.27 ± 290.89 μmol/L, *p* < 0.001, Cohen's *d* = 12.428), and calcium levels (2.22 ± 0.19 vs. 2.09 ± 0.22 mmol/L, *p* < 0.001, Cohen's *d* = 0.272) were higher in the hemodialysis group. No significant differences were found in other laboratory indicators between the two groups.

**Table 1:** Comparison of demographic and laboratory data between the hemodialysis and pre‐dialysis groups (*n* = 250).

Parameters	Hemodialysis group (*n* = 153)	Pre‐dialysis group (*n* = 97)	*t/Z/χ* ^*2*^	*p*	Cohen's *d*/Cramer's *V*
Gender, male [*n*(%)]	99 (64.7%)	55 (56.7%)	1.608	0.205	0.080
Age (years)	63.9 ± 15.2	63.2 ± 15.0	−0.371	0.711	0.187
BMI (kg/m^2^)	22.36 ± 3.14	23.77 ± 3.64	2.857	0.005	−0.771
Hypertension [*n*(%)]	68 (44.4%)	37 (38.1%)	0.967	0.325	0.067
Diabetes [*n*(%)]	139 (90.8%)	84 (86.6%)	1.114	0.291	0.062
High‐sensitivity C‐reactive protein (mg/L)	1.00 (0.50, 4.83)	1.41 (0.50, 9.31)	−1.244	0.214	−0.883
White blood cell count (× 10^3^/mm^3^)	5.62 ± 1.63	6.49 ± 1.93	3.828	< 0.001	−0.659
Neutrophil percentage (%)	67.43 ± 8.10	70.73 ± 9.41	2.941	0.004	−1.123
Lymphocyte percentage (%)	20.13 ± 6.39	17.73 ± 7.05	−2.7	0.007	0.931
Hemoglobin (g/L)	109.54 ± 15.17	87.38 ± 22.44	−8.563	< 0.001	5.225
Platelet (× 10^3^/mm^3^)	169.37 ± 61.55	192.88 ± 76.12	2.682	0.008	−2.869
Albumin (g/L)	36.81 ± 4.34	33.53 ± 5.16	−5.268	< 0.001	1.524
Globulin (g/L)	29.07 ± 5.72	27.99 ± 4.75	−1.493	0.137	0.466
Urea nitrogen (mmol/L)	22.17 ± 6.98	25 ± 11.54	2.067	0.041	−0.962
Creatinine (μmol/L)	810.35 ± 251.91	607.27 ± 290.89	−5.845	< 0.001	12.428
Uric acid (μmol/L)	397.35 ± 101.85	438.16 ± 152.08	2.332	0.021	−3.705
Triglyceride (mmol/L)	1.63 ± 1.03	1.69 ± 0.96	0.434	0.665	−0.058
Total cholesterol (mmol/L)	3.45 ± 1.01	4.18 ± 1.46	4.128	< 0.001	−0.667
High density lipoprotein cholesterol (mmol/L)	1.02 ± 0.38	1.06 ± 0.33	0.890	0.374	−0.072
Low density lipoprotein cholesterol (mmol/L)	2.16 ± 0.74	2.62 ± 1.03	3.649	< 0.001	−0.493
Calcium (mmol/L)	2.22 ± 0.19	2.09 ± 0.22	−4.560	< 0.001	0.272
Phosphorus (mmol/L)	1.64 ± 0.55	1.74 ± 0.59	1.324	0.187	0.134
Parathyroid hormone(nmol/L)	16.56 (7.37, 8.57)	19.00 (12.90, 25.30)	−1.266	0.221	0.260

Abbreviation: BMI, body mass index.

Of the patients with hemodialysis, 21 received conventional hemodialysis while the remainder underwent combined hemodialysis and hemofiltration. Regarding dialyzer types, 10 patients used low‐flux dialyzers compared with 87 using high‐flux dialyzers. The treatment frequency distribution showed 8 patients receiving dialysis twice weekly versus 89 patients undergoing three sessions per week. All treatment sessions maintained a standard duration of 4 h. The dialysate composition was standardized at 2.0 mmol/L potassium and 1.5 mmol/L calcium concentrations. The cohort demonstrated a mean *Kt*/*V* of 1.49 ± 0.21, indicating adequate dialysis adequacy. Importantly, comparative analysis revealed no statistically significant differences in any of these dialysis parameters between patients with treatment durations ≤ 12 months versus those > 12 months.

### 3.2 Comparison of T Lymphocyte Subsets and Mitochondrial Parameters

Comparison of T lymphocyte subsets between the hemodialysis and pre‐dialysis groups revealed a significant increase in the percentage of CD45+ lymphocytes in the hemodialysis group (20.13 ± 7.15 vs. 17.74 ± 7.28, *p* = 0.011, Cohen's *d* = 0.889). No significant differences were observed in other T lymphocyte subset indicators between the two groups. Analysis of T lymphocyte mitochondrial parameters showed that the MM of CD3+ (2.66 ± 1.03 vs. 3.18 ± 1.45, *p* = 0.001, Cohen's *d* = ‐0.476), CD4+ (2.96 ± 1.17 vs. 3.56 ± 1.53, *p* = 0.001, Cohen's *d* = ‐0.528), and CD8+ T lymphocytes (2.28 ± 1.22 vs. 2.81 ± 1.64, *p* = 0.007, Cohen's *d* = ‐0.449), as well as the MMP‐Low% of CD3+ (21.86 ± 10.60 vs. 26.97 ± 11.76, *p* = 0.001, Cohen's *d* = ‐1.539), CD4+ (15.53 ± 9.78 vs. 20.66 ± 10.42, *p* < 0.001, Cohen's *d* = ‐1.621), and CD8+ T lymphocytes (31.12 ± 13.25 vs. 35.28 ± 15.17, *p* = 0.024, Cohen's *d* = ‐1.110), were significantly lower in the hemodialysis group (Table 2). While the T lymphocytes MM differences were statistically significant (*p* < 0.05), their practical impact appeared limited (Cohen's *d* = 0.449–0.528). Conversely, the MMP‐Low% values for all T lymphocytes subsets showed large effect sizes (*d* > 1.0), with CD4+ T cells exhibiting the most dramatic reduction (*d* = 1.621), supporting mitochondrial dysfunction as a key driver of immune abnormalities in hemodialysis.

**Table 2:** Comparison of T lymphocyte subsets and mitochondrial parameters between the hemodialysis and pre‐dialysis groups (*n* = 250).

Parameters	Hemodialysis group (*n* = 153)	Pre‐dialysis group (*n* = 97)	*t/Z/χ* ^*2*^	*p*	Cohen's *d*/Cramer's *V*
CD45 + lymphocyte absolute count (/μL)	1006.21 ± 429.62	1028.82 ± 461.03	0.394	0.694	−1.076
CD3 + T lymphocyte absolute count (/μL)	677.94 ± 307.80	699.12 ± 315.59	0.525	0.600	−1.202
CD4 + T lymphocyte absolute count (/μL)	401.40 ± 184.96	428.91 ± 206.42	1.095	0.274	−1.979
CD8 + T lymphocyte absolute count (/μL)	219.35 ± 135.03	217.42 ± 136.59	−0.110	0.913	0.166
CD4 + PD‐1 + T lymphocyte absolute count (/μL)	94.41 ± 54.41	101.31 ± 56.83	0.960	0.338	−0.927
CD8 + PD‐1 + T lymphocyte absolute count (/μL)	51.35 ± 37.32	50.90 ± 41.74	−0.088	0.930	0.072
Percentage of CD45+lymphocytes (%)	20.13 ± 7.15	17.74 ± 7.28	−2.553	0.011	0.889
Percentage of CD3 + T lymphocytes (%)	67.56 ± 9.66	67.94 ± 10.33	0.293	0.77	−0.120
Percentage of CD4 + T lymphocytes (%)	40.25 ± 8.28	41.89 ± 9.42	1.448	0.149	−0.556
Percentage of CD8 + T lymphocytes (%)	21.90 ± 8.87	21.16 ± 8.24	−0.661	0.509	0.252
Percentage of CD4 + PD‐1 + T lymphocytes (%)	24.18 ± 9.12	24.79 ± 9.67	0.508	0.612	−0.202
Percentage of CD8 + PD‐1 + T lymphocytes (%)	24.24 ± 11.06	23.71 ± 10.69	−0.380	0.704	0.163
PCD4 + /CD8 + T lymphocytes ratio	2.21 ± 1.14	2.36 ± 1.30	0.960	0.338	−0.137
CD3 + T lymphocytes MM	2.66 ± 1.03	3.18 ± 1.45	3.315	0.001	−0.476
CD4 + T lymphocytes MM	2.96 ± 1.17	3.56 ± 1.53	3.319	0.001	−0.528
CD8 + T lymphocytes MM	2.28 ± 1.22	2.81 ± 1.64	2.728	0.007	−0.449
CD3 + T lymphocytes MMP‐low%	21.86 ± 10.60	26.97 ± 11.76	3.479	0.001	−1.539
CD4 + T lymphocytes MMP‐low%	15.53 ± 9.78	20.66 ± 10.42	3.885	< 0.001	−1.621
CD8 + T lymphocytes MMP‐low%	31.12 ± 13.25	35.28 ± 15.17	2.275	0.024	−1.110

Abbreviations: MM, mitochondria mass; MMP, mitochondrial membrane potential.

The median dialysis duration of patients in the hemodialysis group was 23.0 (9.2, 41.8) months. Based on dialysis duration, patients were divided into those with dialysis ≤ 12 months and those with > 12 months. Comparison of T lymphocyte subsets and mitochondrial indexes between these groups revealed that the absolute count of CD4+ T lymphocytes was significantly lower in patients with dialysis duration > 12 months compared to those with ≤ 12 months(455.66 ± 232.80 vs. 375.06 ± 150.99, *p* = 0.029, Cohen's *d* = 6.049). There were no significant differences in T lymphocyte MM between the two groups; however, the MMP‐Low% of CD3+ (25.17 ± 14.08 vs. 20.25 ± 8.02, *p* = 0.025, Cohen's *d *= 1.557), CD4+ (18.30 ± 13.47 vs. 14.18 ± 7.06, *p* = 0.046, Cohen's *d* = 1.363), and CD8+ T lymphocytes (35.22 ± 16.09 vs. 29.13 ± 11.19, *p* = 0.019, Cohen's *d* = 1.702) were significantly reduced in patients with dialysis duration > 12 months (Table 3). These data further demonstrate that mitochondrial function progressively deteriorates with longer dialysis duration.

**Table 3:** Comparison of T lymphocyte subsets and mitochondrial indexes in hemodialysis patients with different dialysis durations (*n* = 153).

Parameters	Dialysis duration ≤ 12 months (*n* = 50)	Dialysis duration > 12 months (*n* = 103)	*t/Z/χ* ^*2*^	*p*	Cohen's *d*
CD45+ lymphocyte absolute count (/μL)	1101.5 ± 533.05	959.95 ± 363.24	1.696	0.094	6.921
CD3 + T lymphocyte absolute count (/μL)	751.9 ± 377.58	642.03 ± 262.12	1.852	0.068	6.348
CD4 + T lymphocyte absolute count (/μL)	455.66 ± 232.80	375.06 ± 150.99	2.231	0.029	6.049
CD8 + T lymphocyte absolute count (/μL)	229.53 ± 156.18	214.41 ± 124.00	0.649	0.518	1.304
CD4 + PD‐1 + T lymphocyte absolute count (/μL)	97.74 ± 50.98	92.79 ± 56.17	0.527	0.599	0.671
CD8 + PD‐1 + T lymphocyte absolute count (/μL)	52.98 ± 37.61	50.56 ± 37.34	0.375	0.708	0.396
Percentage of CD45+lymphocytes (%)	20.88 ± 8.21	19.77 ± 6.60	0.896	0.372	0.414
Percentage of CD3 + T lymphocytes (%)	68.32 ± 9.40	67.2 ± 9.81	0.674	0.501	0.362
Percentage of CD4 + T lymphocytes (%)	41.65 ± 8.91	39.57 ± 7.91	1.465	0.145	0.726
Percentage of CD8 + T lymphocytes (%)	21.05 ± 8.93	22.31 ± 8.85	−0.818	0.415	−0.420
Percentage of CD4 + PD‐1 + T lymphocytes (%)	22.89 ± 9.58	24.8 ± 8.87	−1.213	0.227	−0.631
Percentage of CD8 + PD‐1 + T lymphocytes (%)	24.13 ± 12.34	24.3 ± 10.45	−0.086	0.931	−0.050
PCD4 + /CD8 + T lymphocytes ratio	2.44 ± 1.35	2.10 ± 1.01	1.572	0.120	0.320
CD3 + T lymphocytes MM	2.58 ± 0.81	2.70 ± 1.12	−0.704	0.482	−0.124
CD4 + T lymphocytes MM	2.99 ± 1.36	2.94 ± 1.07	0.248	0.804	0.046
CD8 + T lymphocytes MM	2.21 ± 1.15	2.31 ± 1.26	−0.502	0.616	−0.096
CD3 + T lymphocytes MMP‐Low%	25.17 ± 14.08	20.25 ± 8.02	2.298	0.025	1.557
CD4 + T lymphocytes MMP‐low%	18.30 ± 13.47	14.18 ± 7.06	2.032	0.046	1.363
CD8 + T lymphocytes MMP‐low%	35.22 ± 16.09	29.13 ± 11.19	2.406	0.019	1.702

Abbreviations: MM, mitochondria mass; MMP, mitochondrial membrane potential.

### 3.3 Correlation Analysis of T Lymphocyte Mitochondrial Parameters

In the final models, hemodialysis was significantly associated with reduced mitochondrial parameters after adjusting for the aforementioned covariates: (1) CD3+ T lymphocyte MM decreased by 0.699 units (*β* = −0.699, 95% confidence interval [CI]: −1.167 to −0.232, *p* = 0.004, adjusted *R* ^2^ = 0.129); (2) CD4+ T lymphocyte MM decreased by 0.735 units (*β* = −0.735, 95%CI: −1.192 to −0.277, *p* = 0.002, adjusted *R* ^2^ = 0.156); (3) CD8+ T lymphocyte MM decreased by 0.846 units (*β* = −0.846, 95%CI: −1.370 to −0.323, *p* = 0.002, adjusted *R* ^2^ = 0.132); (4) CD3+ T lymphocyte MMP‐Low% decreased by 4.374% (*β* = −4.374, 95%CI: −8.349 to −0.398, *p* = 0.031, adjusted *R* ^2^ = 0.155); (5) CD4+ T lymphocyte MMP‐Low% decreased by 4.630% (*β* = −4.630, 95%CI: −8.448 to −0.812, *p* = 0.018, adjusted *R* ^2^ = 0.134) (Table 4).

**Table 4:** Linear regression analysis of mitochondrial parameters of T lymphocytes (*n* = 250).

Parameters	Model 1		Model 2		Model 3	
	*β* ± SE (95%CI)	*p*	*β* ± SE (95%CI)	*p*	*β* ± SE (95%CI)	*p*
CD3+ T lymphocytes MM	−0.519 ± 0.157 (−0.828, −0.211)	0.001	−0.546 ± 0.154 (−0.849, −0.242)	< 0.001	−0.699 ± 0.237 (−1.167, −0.232)	0.004
CD4+ T lymphocytes MM	−0.605 ± 0.172 (−0.943, −0.267)	0.001	−0.637 ± 0.17 (−0.973, −0.302)	< 0.001	−0.735 ± 0.232 (−1.192, −0.277)	0.002
CD8+ T lymphocytes MM	−0.529 ± 0.182 (−0.886, −0.171)	0.004	−0.561 ± 0.179 (−0.913, −0.209)	0.002	−0.846 ± 0.265 (−1.37, −0.323)	0.002
CD3+ T lymphocytes MMP‐low%	−5.115 ± 1.436 (−7.943, −2.286)	< 0.001	−5.061 ± 1.393 (−7.806, −2.317)	< 0.001	−4.374 ± 2.014 (−8.349, −0.398)	0.031
CD4+ T lymphocytes MMP‐low%	−5.133 ± 1.303 (−7.699, −2.568)	< 0.001	−5.053 ± 1.269 (−7.552, −2.553)	< 0.001	−4.630 ± 1.934 (−8.448, −0.812)	0.018
CD8+ T lymphocytes MMP‐low%	−4.154 ± 1.826 (−7.749, −0.558)	0.024	−4.018 ± 1.757 (−7.479, −0.557)	0.023	−1.901 ± 2.487 (−6.811, 3.009)	0.446

*Note:* Model 1: single‐factor linear regression analysis, uncorrected. Model 2: multi‐factor linear regression analysis, corrected for gender, age, and diabetes. Model 3: multivariate linear regression analysis, adjusted for gender, age, diabetes, BMI, white blood cell count, hemoglobin, albumin, and low‐density lipoprotein cholesterol. Abbreviations: BMI, body mass index; MM, mitochondria mass; MMP, mitochondrial membrane potential.

## 4 Discussion

In this study, we compared T lymphocyte subsets and mitochondrial parameters between ESRD patients on hemodialysis and those not undergoing dialysis. The results indicated that the percentage of CD45+ total lymphocytes in the peripheral blood of hemodialysis patients was higher compared with pre‐dialysis patients, consistent with the increased lymphocyte proportion observed in routine blood tests. However, as dialysis duration extended, the absolute count of CD4+ T lymphocytes decreased. These findings suggest that despite an increased lymphocyte proportion in hemodialysis patients, their immune function deteriorates with prolonged dialysis. Additionally, MM and the percentage of cells with low MMP (MMP‐Low%) in CD3+, CD4+, and CD8+ T lymphocytes were significantly reduced in hemodialysis patients. Linear regression analysis, after adjusting for various factors, revealed that hemodialysis was independently correlated with T lymphocyte MM and MMP‐Low% in CD3+ and CD4+ T lymphocytes. Notably, the adjusted *R* ^2^ values for the final models ranged from 0.129 to 0.156, accounting for approximately 13%–16% of the variance in mitochondrial parameters. While the majority of the variance remains unexplained by the included variables, the combination of statistically significant associations (all *p* < 0.05) and moderate‐to‐large effect sizes (Cohen's d) provides robust empirical support for the core hypothesis that hemodialysis is independently associated with T cell mitochondrial dysfunction.

Previous studies have confirmed that various immune cells and cytokines are involved in the occurrence and development of CKD, with most CKD patients exhibiting severe T lymphocyte subset imbalances and dysfunction [10]. Immune system disorders characterized by immune insufficiency and chronic inflammation are present in ESRD patients, regardless of dialysis status [11]. ESRD patients have significantly fewer T lymphocytes, CD4+, and CD8+ T cells compared ‐with healthy individuals [12]. In ESRD patients, oxidative stress, uremia, secondary hyperparathyroidism, iron load, and inflammation collectively lead to increased apoptosis of naive and central memory CD4+ and CD8+ T cells [13]. Studies have shown that the inflammatory environment induced by uremia contributes to premature aging of T lymphocytes, resulting in loss of naive T cells and T cell immune deficiency [14]. T lymphocytes are central to the production of adaptive immune responses; disruption in the balance of T cell subsets directly reduces immune function and increases infection risk [15]. Recent studies have found that low levels of CD3+, CD4+, and CD8+ T lymphocyte subsets significantly increase the risk of adverse renal outcomes and infection [3]. However, few studies have evaluated the effects of hemodialysis on T lymphocyte subsets in ESRD patients. This study found that compared with pre‐dialysis ESRD patients, the proportion of total lymphocytes in hemodialysis patients increased, but there were no significant changes in the count and proportion of lymphocyte subsets. Nonetheless, with the extension of dialysis duration, the absolute count of CD4+ lymphocytes decreased significantly, indicating further decline in immune function with prolonged dialysis. Chronic inflammation, often present in hemodialysis patients due to uremic toxins, bacterial products, repeated infections, and biocompatibility reactions, can lead to chronic stimulation of lymphocytes, triggering immune deficiency and increasing susceptibility to infection [16].

Numerous studies have shown that cell damage, inflammation, and fibrosis are all caused by mitochondrial dysfunction. The primary function of mitochondria is to perform oxidative phosphorylation and produce ATP to meet cellular energy demands [17]. Oxidative phosphorylation is a highly efficient ATP‐producing mechanism that uses electron transport chains driven by the oxidation of various substrates [18]. The movement of electrons along the mitochondrial electron transport chain is linked to the transport of protons across the mitochondrial membrane, creating an electrochemical gradient known as MMP, which is used to phosphorylate adenosine diphosphate (ADP) to ATP. MMP is a core bioenergy parameter that regulates the production of ROS [19]. Changes in MMP or the ROS clearance system affect mitochondrial ROS production [20]. ROS at physiological levels serve important signaling functions, such as inducing cell proliferation and survival under stress conditions [21, 22]. However, excessive mitochondrial ROS can be pathogenic and cause cellular oxidative damage [23]. Overproduction of mitochondrial ROS is associated with mitochondrial dysfunction, leading to cell damage and disease progression. Thus, mitochondria are crucial for kidney function, an organ with high energy requirements and abundant mitochondria. Despite this, few studies have analyzed mitochondrial damage in T lymphocytes in ESRD patients. Mitochondria also play a central role in T cell energy metabolism and cell homeostasis, providing ATP and regulating metabolic activity within cells. They are essential for inducing and regulating immune responses [6]. T cell activation increases MM, MMP, and mitochondrial DNA levels [24, 25]. Mitochondria play an anabolic role in proliferation and serve as a signaling platform that coordinates T cell activation and differentiation [26]. Studies have found that differences in mitochondrial function are related to dynamic changes in mitochondrial morphology [27]. Mitochondria can alter their morphology and position in cells through coordinated fission and fusion cycles to regulate their function and cellular metabolism [28]. MMP‐Low is associated with ATP production capability [29]. An appropriate MMP‐Low level is essential for maintaining mitochondrial oxidative phosphorylation to produce ATP, which supports the normal physiological function of immune cells. Conversely, excessively low MMP‐Low levels exacerbate oxidative stress. MMP‐Low has been reported clinically, and it is more closely associated with the disease [30, 31]. In this study, T cells from hemodialysis patients exhibited abnormal mitochondrial metabolism and decreased MM. The mitochondrial structure and function of T lymphocytes were more severely impaired in hemodialysis patients compared to pre‐dialysis patients.

Mitochondria are highly plastic organelles that continuously alter their shape and size through fusion and fission processes in response to metabolic and signaling cues from the cellular environment. They house all mechanisms essential for cellular respiration, with larger MM linked to enhanced recall ability, increased oxidative phosphorylation, and prolonged cell longevity. Research has shown that MM decreases with age [32]. Additionally, CD4+ T lymphocytes have been observed to possess larger MM and exhibit greater oxidative capacity than CD8+ T cells, enabling them to maintain effector functions [33]. Aging results in CD8+ T lymphocytes having reduced MM, impaired mitochondrial biogenesis, and a mitochondrial hypopolarization phenotype compared with CD4+ T lymphocytes, suggesting more severe mitochondrial damage in CD8+ T lymphocytes [34]. This study found that CD4+ T cells in ESRD patients also exhibited larger MM compared to CD8+ T lymphocytes. Furthermore, hemodialysis patients had lower MM than pre‐dialysis patients. After adjusting for age and other confounding factors, hemodialysis was independently associated with decreased T lymphocyte MM, indicating more severe mitochondrial damage in hemodialysis patients compared with pre‐dialysis ESRD patients, unaffected by factors such as age.

The observed biochemical differences between hemodialysis and pre‐dialysis groups may have important implications for T lymphocyte mitochondrial function. The hemodialysis group showed significantly lower white blood cell counts, neutrophil percentages, platelet counts, uric acid, and lipid profiles, along with higher lymphocyte percentages, hemoglobin, albumin, creatinine, potassium, and calcium levels. These alterations may reflect multiple interconnected pathways affecting immune cell metabolism: First, the efficient clearance of uremic toxins through dialysis (as evidenced by lower uric acid) may paradoxically create a new metabolic milieu where residual protein‐bound toxins continue to induce mitochondrial oxidative stress in lymphocytes. Second, the combination of lower BMI/cholesterol with higher albumin suggests a protein‐energy wasting state that could force lymphocytes to adapt their mitochondrial metabolic pathways. Third, elevated calcium and potassium levels in hemodialysis patients may directly impact mitochondrial membrane potential and calcium‐dependent apoptotic pathways in T lymphocytes. These biochemical alterations should be interpreted in conjunction with our mitochondrial parameter measurements, as they may collectively contribute to the observed T lymphocyte dysfunction in ESRD patients. Future studies should examine whether these parameter changes correlate with specific T lymphocyte subset alterations and mitochondrial functional readouts.

This study has several limitations. First, the retrospective cross‐sectional design inherently limits causal interpretation and may introduce selection bias. Second, although our sample size met statistical requirements, larger multicenter cohorts could enhance the generalizability of findings. Third, unmeasured confounders including pharmacotherapy, systemic inflammation, and nutritional status require dedicated evaluation in subsequent research. Fourth, most critically, the clinical relevance of observed mitochondrial abnormalities necessitates functional validation through T lymphocyte activation assays. Collectively, these limitations highlight the imperative need for prospective longitudinal studies incorporating multimodal biomarker assessment and mechanistic investigations. In addition, our study elucidates novel mechanistic evidence of mitochondrial dysfunction in ESRD patients' T lymphocytes, with particular focus on MM and MMP‐Low% parameters, and these findings may establish two critical translational foundations: (1) Quantifiable mitochondrial parameters that may serve dual roles as both therapeutic targets and treatment response biomarkers; (2) A conceptual framework to guide development of mitochondria‐targeted immunotherapies. We propose these mechanistic insights should inform subsequent therapeutic development strategies.

## 5 Conclusion

In summary, compared with pre‐dialysis ESRD patients, MM and MMP‐Low% of T lymphocytes were significantly reduced in hemodialysis patients. Hemodialysis was found to be independently correlated with MM of T lymphocytes and MMP‐Low% of CD3+ and CD4+ T lymphocytes. The results indicate significant differences in T lymphocyte MM and MMP‐Low% between hemodialysis and pre‐dialysis ESRD patients, with mitochondrial dysfunction being more pronounced in hemodialysis patients. Hemodialysis does not appear to improve T lymphocyte mitochondrial function.

## Author Contributions

**Chunlei Luo:** funding acquisition, methodology, writing – original draft. **Sizeng Bao:** project administration, writing – review and editing. **Xueyan Bian:** funding acquisition, project administration, writing – review and editing. **Lingling Bao:** data curation, formal analysis, writing – review and editing. **Chunyang Ji:** data curation, formal analysis, writing – review and editing. **Jianwei Ma:** writing – review and editing. **Jiancheng Huang:** writing – review and editing.

## Disclosure

The authors declare no conflicts of interest.

## Ethics Statement

This study was according to the principles of the Declaration of Helsinki and approved by the Ethics Committee of the First Affiliated Hospital of Ningbo University. No intervention measures were taken to the patients during the study, which did not harm the interests of the patients, and the exemption from informed consent was applied and passed.

## Data Availability

The first author can provide summary data that were used for this study upon reasonable request.
